# How Hydrogen Dielectric Strength Forces the Work Voltage in the Electric Discharge Machining

**DOI:** 10.3390/mi9050240

**Published:** 2018-05-15

**Authors:** José António Almacinha, António Mendes Lopes, Pedro Rosa, José Duarte Marafona

**Affiliations:** 1 Departamento de Engenharia Mecânica, Faculdade de Engenharia da Universidade do Porto, Rua Roberto Frias, 4200-465 Porto, Portugal; jasa@fe.up.pt (J.A.A.); aml@fe.up.pt (A.M.L.); 2 Instituto de Engenharia Mecânica (IDMEC), Instituto Superior Técnico, Universidade de Lisboa, Av. Rovisco Pais N1, 1049-001 Lisboa, Portugal; pedro.rosa@tecnico.ulisboa.pt

**Keywords:** electric discharge machining (EDM), dielectric strength, work voltage control, discharge mechanism, multiple discharges, performance

## Abstract

An electro-thermal model based on the Joule heating effect is proposed to simulate a single discharge in an electric discharge machining process. Normally, the dielectric strength of the hydrocarbons oil is approximately 20 MV/m, but it varies with both the thickness of the film and its decomposition. After the breakdown, the hydrocarbon oil has an average dielectric strength value of 2 MV/m. This value is close to the dielectric strength of the hydrogen, which is the main gas that results from the hydrocarbon oil decomposition, at temperatures between 6000 K and 9000 K. Therefore, the electric discharge occurs in a hydrogen atmosphere that imposes both the discharge gap and the work voltage. A 200 V voltage is associated to a 100 μm discharge gap, leading to a 20 V work voltage. Therefore, the 3 V work voltage control corresponds to approximately 15 μm. In other words, the increase of the discharge gap originates other discharge during the discharge pulse. The work voltage control, together with the multiple discharge method, is taken into account. The 100 μm discharge gap corresponds to the higher value of the transitory discharge gap that over evaluates the material removal and the tool wear rates. The results of the numerical simulations are validated with experimental data.

## 1. Introduction

Electric discharge machining (EDM) has been a well-known process for more than 50 years. Nowadays, it is widely used in a large number of industrial areas, namely in molds and aeronautics. The EDM consists in removing small volumes of material, which are molten or vaporized, by means of current discharges, or sparks, between two electrodes (the tool and the workpiece). The sparks are created in a flowing dielectric fluid, generally oil, that guarantees a high plasma pressure and a high removing force on the molten metal when the plasma collapses [1]. The EDM is governed by an electro-thermal phenomenon. However, the electric potential gradient is the removing force on the molten material, being exerted during the electric discharge.

Many studies [2,3,4,5,6] address the process of material removing in EDM. Yeo et al. [7] show that the molten material is removed mainly due to the effect of fluid flushing. This exerts a force on the molten material, which is more effective for high current discharges. However, in the present research we demonstrate that the electric potential gradient, as an electric force, is more significant when compared to fluid flushing, because the electric force is exerted during the electric discharge, while the fluid flushing only acts at the end of discharge pulse.

In previous studies [2,3,4,5,6,8,9], the temperature and the power sources are of various types. Accordingly, point, disk, and Gaussian heat sources are applied on the surface of the workpiece and tool, which are in contact with a discharge channel. There is an exchange of heat by radiation between the discharge channel and the surrounding dielectric, which is at ambient temperature. The radius of the discharge channel depends on the discharge current and, also, on the time needed to establish the electric discharge. In other words, it depends on the ignition delay. Therefore, in this paper we consider the discharge channel constant. For the applied current intensities, the discharge gap is also constant, due to an applied voltage of 200 V and a 2 MV/m dielectric strength, yielding the work voltage of 20 V.

The maximum temperature is obtained at the center of the gap between the electrodes [10,11]. Therefore, the models [2,3,4,5,6] do not take into account the effect of maximum temperature propagation caused by the increase of the discharge gap during the discharge pulse. In the present research we consider this phenomenon as transitory. The gap is variable, meaning that its reference value changes, which leads the electrodes to new positions that can stay closer to, or far away from, the center of maximum temperature.

In the present research, it is demonstrated that the energy used in a single discharge is approximately 35% of the pulse energy, which depends on the discharge current and the work voltage. The crater may be created with a depth that can lead to multiple discharges. The total number of discharges depends on both the crater depth and the value of the work voltage control. Moreover, the work voltage control (3 V) depends on the work voltage (20 V) that is associated with a 100 μm gap width, which is influenced by the dielectric strength. Therefore, the sum of depths of the erosion cavities obtained on the surfaces of the tool and the workpiece that give origin to other discharges is equal to 15 μm. Additionally, if the sum of depths of erosion cavities is equal to, or greater than, 45 μm, then a maximum of three discharges (35 × 3) may occur during the discharge pulse. The work voltage control value is 20 ± 3 V and is compared with the feedback gap voltage. The difference between the two values drives the servomechanism to maintain the 20 V work voltage. Moreover, a 200 V voltage is applied to the electrodes, which falls to the work voltage of 20 V when the current passes through the formed discharge channel, and an electric potential gradient of 200 V is established between electrodes.

To sum up, the hydrogen dielectric strength forces the discharge gap and work voltage according to the applied voltage. The material removal rate (MRR), the tool wear rate (TWR) and the average maximum height of the work piece surface roughness profile (*R_z_*) are computed using a finite element model with the effect of multiple discharges.

## 2. Mathematical Modelling

The finite element analysis (FEA) is carried on with the ABAQUS/Standard Code [12], which allows for a coupled electro-thermal analysis of the EDM.

The Joule heating effect arises when the electric energy flowing through a conductor is converted into thermal energy. The coupling has two sources: the dependency between the electric conductivity and the temperature, and the relationship between the generated internal heat and the electric current. However, in our analysis the electric conductivity is considered independent of the temperature. Moreover, the thermal problem includes the heat conduction through the electrodes, and the heat exchange by radiation between the discharge channel and the surrounding dielectric, which is considered at the ambient temperature (288 K). We do not consider the forced heat convection caused by the fluid flowing through the mesh. The electro-thermal elements have both temperature and electrical potential as nodal variables.

### 2.1. Governing Equation

The governing equation of the phenomenon is based on the Maxwell’s equation of conservation of charge in a conducting material. Assuming that there is a steady-state direct current, the equation reduces to

(1) $$\int_{S}J\cdot ndS=\int_{V}r_{c}dV$$

Introducing an arbitrary variational, that is, the electric potential field, *δφ*, and integrating over the volume, we have

(2) $$-\int_{V}\frac{\partial \delta \varphi }{\partial x}JdV=\int_{S}\partial \varphi JdS+\int_{V}\partial \varphi r_{c}dV$$

where J, defined as −J·n, is the density of current entering the control volume (*V*) across the surface (*S*), and $r_{c}$ is the internal volumetric current source per unit volume. Equation (2) is the governing model of the coupled electro-thermal problem.

### 2.2. Constitutive Behavior

The flow of the electrical current is described by the Ohm’s law

(3) $$J=\sigma^{E}\cdot E$$

where $\sigma^{E}\left(\theta ,f^{\alpha }\right)$ denotes the electrical conductivity matrix, *θ* is the temperature, *f^α^*, with *α* = 1, 2, …, are any predefined field variables, and *E(x)* is the electric field intensity. The electric conductivity can be isotropic, orthotropic, or fully anisotropic, but in the present analysis we consider the isotropic case.

Introducing the Ohm’s law, the governing equation of charge conservation becomes

(4) $$\int_{V}\frac{\partial \delta \varphi }{\partial x}\cdot \sigma^{E}\cdot \frac{\partial \varphi }{\partial x}dV=\int_{S}\delta \varphi JdS+\int_{V}\delta \varphi r_{c}dV$$

### 2.3. Thermal Energy Balance

The behavior of the heat conduction is described by the basic energetic balance

(5) $$\int_{V}\rho \dot{U}\delta \theta dV+\int_{V}\frac{\partial \delta \theta }{\partial x}\cdot k\cdot \frac{\partial \theta }{\partial x}dV=\int_{V}\delta \theta rdV+\int_{S}\delta \theta qdS$$

where *V* is a volume of solid material, with surface area *S*, *ρ* denotes the density of the material, $\dot{U}$ is the internal energy, *k* represents the thermal conductivity matrix, *q* is the heat flux per unit area of the body, flowing into the body, and *r* denotes the heat generated within the body.

Equations (4) and (5) describe the electric and thermal problems, respectively. Thus, the internal heat generation in the thermal problem is a function of the electric current, $r=r_{ec}\left(J\right)$.

### 2.4. Surface Conditions

The boundary conditions of the surface *S* of the body can be prescribed, *S_p_*, and can include parts that interact with nearby surfaces of other bodies, *S_i_*. Prescribed boundary conditions include the electrical potential, φ=φ(x,t), the temperature, θ=θ(x,t), the electrical current density, J=J(x,t), and the heat flux, q=q(x,t). The surface interaction model includes heat conduction from and through the discharge channel, the workpiece, and the tool. It includes also the heat exchange by radiation between the interface of the discharge channel and the surrounding dielectric. There is an electric current flowing across the interfaces between the discharge channel/workpiece and discharge channel/tool. The surface convection is not included. The model considers the ambient temperature of 288 K. The heat conduction is modelled by

(6) $$q_{c}=k_{g}\left(\theta_{B}-\theta \right)$$

where *θ* is the temperature on the surface of the body under consideration, *θ_B_* is the temperature on the surface of the other body, and $k_{g}\left(\bar{\theta ,}{\bar{f}}^{\alpha }\right)$ is the gap thermal conductivity.

The electric current flowing between the interface surfaces is modelled as

(7) $$J=\sigma_{g}\left(\varphi_{B}-\varphi \right)$$

where *φ* is the electrical potential on the surface of the body under consideration, *φ_B_* is the electrical potential on the surface of the other body, and $\sigma_{g}(\bar{\theta },{\bar{f}}^{\alpha }$) is the electric conductivity of the gap. The electric energy dissipated by the current flowing across the interface is given by

(8) $$P_{ec}=J\left(\varphi_{B}-\varphi \right)=\sigma_{g}{\left(\varphi_{B}-\varphi \right)}^{2}$$

being released as heat on the surface of the bodies:

(9) $$q_{ec}=f\eta_{g}P_{ec}$$

and

(10) $$q_{ec}^{B}=\left(1-f\right)\eta_{g}P_{ec}$$

where *η_g_* is an energy conversion factor and *f* specifies how the total heat is distributed between the interface surfaces. A *P_ec_* averaged value over the time increment is used in the transient analysis.

Considering the surface interaction effects and the electric energy released as thermal energy, the governing electric and thermal equations become

(11) $$\int_{V}\frac{\partial \delta \varphi }{\partial x}\cdot \sigma^{E}\cdot \frac{\partial \varphi }{\partial x}dV=\int_{V}\delta \varphi r_{c}dV+\int_{S_{p}}\delta \varphi JdS+\int_{S_{i}}\delta \varphi \sigma_{g}\left(\varphi_{B}-\varphi \right)dS$$

and

(12) $$\int_{V}\rho \dot{U}\delta \theta dV+\int_{V}\frac{\partial \delta \theta }{\partial x}\cdot k\cdot \frac{\partial \theta }{\partial x}dV=\int_{V}\delta \theta rdV+\int_{V}\delta \theta \eta_{g}P_{ec}dV+\int_{S_{p}}\delta \theta qdS+\int_{S_{i}}\delta \theta \left(q_{c}+q_{r}+q_{ec}\right)dS$$

In the Finite Element Method (FEM), the equilibrium is approximated as a finite set of equations by introducing interpolation functions. The discretized quantities represent nodal variables, with nodes shared between adjacent elements. An appropriate interpolation is chosen to provide adequate continuity of the assumed variation. There is an adequate interpolating function for the virtual electric potential and the temperature fields in the thermal problem, transforming the Equations (11) and (12) into a set of discretized electric and thermal expressions.

## 3. Assumptions

### 3.1. Discharge and Plasma Channels

The breakdown of the dielectric oil generates the discharge channel. This is a cylindrical gas channel with a level of ionization capable of conducting the electric current, while maintaining a level of vaporization and ionization during the electric discharge. The pressure inside the discharge channel reaches its maximum immediately after the dielectric breakdown, maintaining this value during the electric discharge. The temperature is quasi constant after the dielectric breakdown and during the electric discharge. Therefore, the thermal properties of the discharge channel are independent of both the temperature and the pressure. We consider also that the radius of the discharge channel is dependent on the discharge current and the small lapse of time necessary to breakdown the dielectric. So, the radius of the discharge channel is constant during the pulse duration, since the discharge current and the ionization time are constant. The discharge gap is considered constant and equal to 100 μm for a work voltage of 20 V and an applied voltage of 200 V. The control of the standard voltage allows an increase of the discharge channel width, which causes a movement of the maximum temperature point in the discharge channel relative to the tool surface, and simultaneously the possibility of other electric discharge to occur.

The radius of the discharge channel is calculated by means of the Equation (13), according to [13],

*R*(*t*) = 2.04 × 10^−3^ × *I*^0.43^ × *t*^0.44^ (13)

where *R*(*t*) is the radius of the discharge channel, *I* denotes the discharge current, and *t* is the lapse of time before the dielectric breakdown or, by other words, the ignition delay. In the present research, this time is considered equal to 500 ns, which corresponds to the time necessary to establish completely the discharge channel.

The electric conductivity is calculated using the Equation (14), as given in [11],

*σ* = (*G* × *I*)/(*π* × *R*^2^ × *E*) (14)

where *σ* is the electric conductivity, *G* is the gap between the electrodes, *I* is the discharge current passing through the discharge channel, *E* is the work voltage equal to 20 V, and *R* is the radius of the discharge channel.

The gap between electrodes is maintained constant and equal to 100 μm, for the discharge currents, by means of the standard voltage control. This is compared with the feedback work voltage, and the difference between the two values is used for driving the servo mechanism, so as to maintain the standard voltage at the level determined by the servo standard voltage control. Moreover, the breakdown of the dielectric oil for all applied currents occurs in similar conditions, namely at the pressure of 10,000 bar and temperature around 6273 K, leading to the thermal properties shown in Table 1, according to Arunachalam et al. [14]. The thermal conductivity is chosen for obtaining the material removal rate for the applied current of 19.3 A, pulse duration of 75 μm, cut depth of 1 mm, and fluid flushing at the pressure of 1 bar.

### 3.2. Heat Distribution

The energy used for removing material from the electrodes is related to the discharge duration, the discharge current and the applied/work voltage or, more precisely, the gap between the electrodes. The amount of heat used in a single discharge is equal to 35% of the energy of the discharge pulse, as pointed out in [15]. However, we postulate that all energy of the discharge pulse can be used in multiple discharges, increasing the material removal rate and, consequently, decreasing the temperature in the discharge channel. The numerical model computes the material removed in a single discharge and the sum of the depths of erosion craters can be greater than 15 μm, originating multiple discharges with depths greater and smaller than those allowed by the control of work voltage. Thus, the amount of energy used in the material removal can be equal to all the energy of the discharge pulse, according to the number of discharges performed.

The study of the cathode, the anode, and the discharge channel in simultaneous originates a natural division of the heat that depends on the thermal properties. Thus, the volume of the crater depends on the input power and the thermal properties of the materials, with the last one depending on the temperature, as shown in [16], Table 2.

### 3.3. Volume of Erosion for a Unique Discharge or Multiple Discharges

The volume of erosion for a single discharge is obtained through the FEM analysis. The material removed of the electrodes is limited by the melting temperature of the materials, which imposes the spherical segment shape for the erosion crater due to the cylindrical energy source. Therefore, the volumes (*V*) removed are calculated using the radius of the molten area (*r*) and its depth (*h*), according to Equation (15):

*V* = *π* × *h* × (3 × *r*^2^ + *h*^2^)/6 (15)

The volume removed of the workpiece divided by the sum of the pulse-on (*t_on_*) and pulse-off (*t_off_*) gives the MRR. The TWR is calculated using the previous strategy applied to the material removed of the tool. The tool wear ratio (TWRa) is calculated by dividing the volume of material removed of the tool by the volume of material removed of the workpiece. The depth of the erosion crater is given in micrometers and represents the average maximum height of the work piece surface roughness profile.

The effect of multiple discharges on the material removal rates of the electrodes is analyzed in this research. Therefore, we postulate a methodology that allows obtaining a correction of the volume removed in a single discharge through the effect of standard voltage control, which induces the occurrence of multiple discharges. Our scheme is related to the standard voltage control (±3 V) through the work voltage (20 V). The work voltage (20 V) imposes a gap between the electrodes of 100 μm and, therefore, the standard voltage control (3 V) allows an increase of the gap width in 15 μm until the occurrence of other discharge.

There is one incomplete discharge when the depth of erosion cavity on the workpiece surface is less than 15 μm. There are multiple discharges when the depth of erosion cavity in the workpiece surface is greater than 15 μm. The number of discharges is calculated by dividing the depth of the erosion cavity in the workpiece surface through 35% of the pulse energy, by the depth of the crater (15 μm) allowed by the standard voltage control (3 V). Moreover, the volume of material removed of the workpiece, during the discharge pulse, corresponds to the volume of a single discharge through the use of 35% of pulse energy multiplied by the number of multiple discharges. The single discharge crater can have a depth equal, greater, or smaller, than 15 μm due to the 35% of the pulse discharge energy. However, the depth of the crater necessary to originate other discharge is equal to 15 μm, since the limit of the standard voltage control (3 V) is reached.

The material removed of the electrode tool is related to the increase of the work gap. This is due to the material removed from the workpiece that allows a relative receding movement of the electrode tool, because it stays far away from the maximum temperature of the center of the discharge channel. The number of discharges is found dividing the depth of erosion cavity through 35% of the pulse energy on the workpiece surface, by the depth of the crater allowed by the standard voltage control (3 V). However, the volume of material removed from the electrode tool corresponds to the volume of a single discharge (35%) divided by the number of multiple discharges.

## 4. Experimental Methodology

This methodology was designed and performed in a die-sinking EDM machine AGIE COMPACT 3, from the manufacturer AGIE, Lausanne, Switzerland, equipped with adaptive control facilities. The adaptive control optimization (ACO) system enables the process optimization automatically and it was switched off so that the results can be generalized to different machines. The electrode tool and workpiece materials are electrolytic copper and steel AISI/SAE-D2, from the supplier Thyssen, Queluz, Portugal; respectively. The bar is a parallelepiped with dimensions of 300 mm × 60 mm × 25 mm. The tools used are copper rods with Ø16 mm in diameter and 100 mm in length. The EDM performance is related to the efficiency, which is determined in the EDM process by the MRR and TWR.

The material removal rate and tool wear rate were calculated using the difference of weight before and after machining. The weight of the steel bar and the copper rod were measured five times and this was used as its average value, with different accuracy due to its difference in weight, thus the steel bar was measured in a Kern PLS balance with accuracy of 0.01 g, from Kern, Albstadt, Germany, and the copper rod was measured using a Mettler H31AR balance, with accuracy of 0.1 mg from Mettler Toledo, Porto, Portugal. The weight calculated was converted in volume using the density of the materials. The volume was divided by the time of machining.

The average maximum height of the work piece surface roughness profile was measured five times and the average value calculated using a Hommelewerk T4000 measurement instrument, from Hommelewerk, Villingen-Schwenningen, Germany.

## 5. Numerical Results and Discussion

### Finite Element Model Using Heat Exchange by Radiation

The electro-thermal model uses the heat exchange by radiation between the plasma channel and the surrounding dielectric at the ambient temperature (288 K). The radiation is global using a coefficient of emissivity equal to one, or by other words, the discharge channel is considered a black body. The heat exchange by radiation decreases the temperature in the discharge channel, tending to decrease the electrode erosion rates during the discharge pulse.

The electro-thermal model has, as input parameters, the discharge current intensity, the applied voltage, the work voltage, the pulse duration, and the pause. The values of the applied voltage and work voltage are 200 V and 20 V, respectively. The remaining input parameters are shown in Table 3(a).

The results of the MRR, TWR, TWRa, and average maximum height of the work piece surface roughness profile for the single discharge (35%) are shown in Table 3(a,b). These results were computed with our numerical model. It should be noted that only the average maximum height of the work piece surface roughness profile agrees well with our experimental data and the experimental data from the machine manual.

The results of the numerical model together with the effect of the multiple discharges [17] applied to the output parameters agree well with the experimental data as shown in Table 4(a,b). We verify that the corrected MRR, TWR, and TWRa agree well with our experimental data and the experimental data from the machine manual.

**Figure 1:**
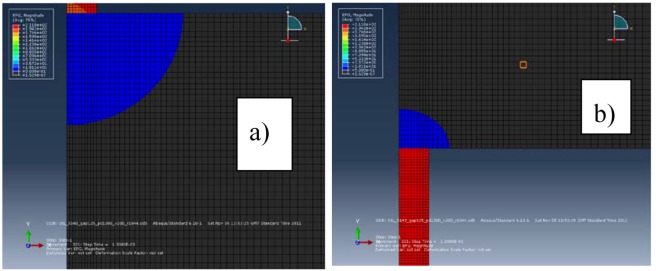
Electrical potential gradient for a current intensity of 51.4 A: (**a**) work piece; (**b**) Tool.

The electric force seems to affect the MRR and the TWR in a similar way, because the error for the MRR is similar to the error for TWR. The effect of the electric force imposed by the electric potential gradient on the tool surface is smaller than the one on the workpiece surface, as shown in Figure 1a,b. The results of the numerical model together with the effect of multiple discharges for the MRR of samples 7, 8, and 9 (see Table 4(a,b)) are higher than the material removed experimentally. This fact can be explained by the action of the electric force at the end of the pulse. All the experiments were done without flushing, while the samples 7.01, 8.01, and 9.01 (see Table 4(a,b)) were carried out with lateral flushing at a pressure of 1 bar. In this case, there is an increase in the material removal rate. This increase is due to the increase in the pressure exerted on the molten material by the lateral flushing at the end of the pulse.

**Figure 2:**
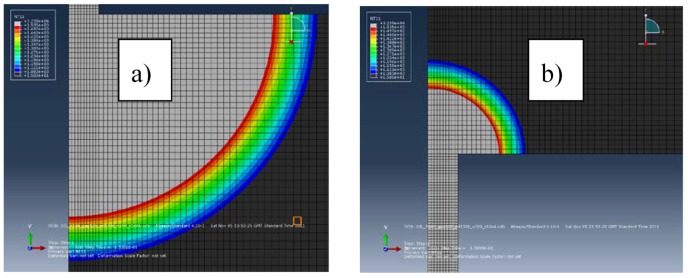
Shape of the erosion crater on the electrodes for a current intensity of 51.4: (**a**) workpiece; (**b**) tool.

**Figure 3:**
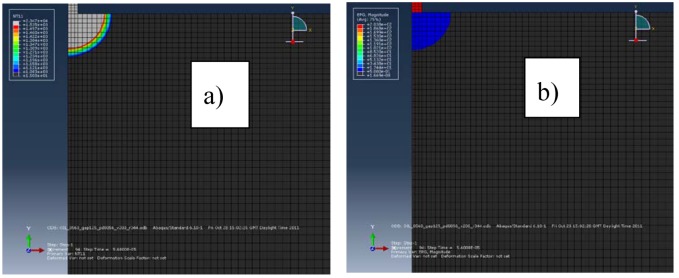
Electric force efficiency for a current intensity of 5.6 A: (**a**) erosion crater; (**b**) electric potential gradient.

Figure 1a,b show the electric potential gradient in the electrodes for the current intensity of 51.4 A. The electric potential gradient generates an electric force on the surface of the electrodes that expulses the molten material during the discharge pulse. The electric potential gradient in the workpiece is higher than in the tool. The area of the molten material ready to be removed of the electrodes, for the current intensity of 51.4 A, can be seen in Figure 2a,b. We verify that it is bigger than the area under the electric potential gradient that generates an electric force. The two areas are very similar for the workpiece and a current intensity of 5.6 A, as can be seen in Figure 3a,b. Therefore, the electric force is more effective for low discharge currents (small pulses) than for high discharge currents (long pulses). All Figures are at the same scale.

The area of the workpiece under the effect of the electric force is close to the molten area for small current intensities, while for high currents the molten area is higher than the area under the electric potential gradient. Therefore, the electric force for high discharge currents is not enough to remove all the molten material. Moreover, there is a high electric force efficiency for small discharge currents, as can be seen in Figure 3a,b.

## 6. Conclusions

From the results of the electro-thermal model, we can conclude that:

(i) The dielectric strength has an important role in the value of the discharge gap and consequently in the work voltage. Thus, a 200 V voltage applied in a dielectric fluid with 20 MV/m dielectric strength leads to 2 MV/m, after its breakdown, imposing a discharge gap of 100 μm and a 20 V work voltage.
(ii) The simulation of the single discharge does not lead to a good prediction of the MRR and the TWR. However, it predicts the average maximum height of the work piece surface roughness profile with good accuracy. The authors did an experimental evaluation of the variation of the material removal rates with the depth of cut. These experimental rates for 1 mm depth are near the rates obtained with the numerical model. Moreover, the results provided by the manufacturer AGIE for the discharge currents, from 5.6 A up to 19.3 A, agree well with the results of our numerical model.
(iii) The occurrence of multiple discharges affects the MRR and the TWR during the machining. Adding this effect on the volume removed of a single discharge yields predicted values of the MRR and TWR very close to the experimental data.
(iv) This electro-thermal model together with the effect of multiple discharges is a good approach for the electric discharge phenomenon in the EDM process. The results of the model show a high degree of agreement with the experimental data.

Thus, it is concluded that the effect of control reference voltage on the volumes removed of the electrodes is very important.

## Figures and Tables

**Table 1:** Properties of the dielectric.

Temperature (°C)	*ρ* (kg/m^3^)	*Cp* (J/kg·°C)	*k* (W/m·°C)
6000	271	7443	160

**Table 2:** Properties of the material of the electrodes.

Properties of Wrought Iron, 0.5% Carbon				Properties of Pure Copper			
Temperature (°C)	*ρ* (kg/m^3^)	*Cp* (J/kg·°C)	*k* (W/m·°C)	Temperature (°C)	*ρ* (kg/m^3^)	*Cp* (J/kg·°C)	*k* (W/m·°C)
20	7,849	460	59	20	8954	383	386
100	--	--	57	100	--	--	379
200	--	--	52	200	--	--	374
300	--	--	48	300	--	--	369
400	--	--	45	400	--	--	363
600	--	--	36	600	--	--	353
800	--	--	33	1083 (melting)	--	--	--
1000	--	--	33	--	--	--	--
1200	--	--	33	--	--	--	--
1535 (melting)	--	--	--	--	--	--	--

**Table 3:** (**a**) Comparison of the results of the electrical-thermal model for a single discharge and experimental data; (**b**) Comparison of the results of the electrical-thermal model for a single discharge and experimental data.

**Machining Conditions**				**Material Removal Rate (MRR) (mm^3^/min)**		
Exp.^t^	Discharge current (A)	Time-on (μs)	Time-off (μs)	Our exp.^ts^	Our numerical model	Agie Sit
1	5.6	56	75	1.45	1.65	0.8
2	8.5	75	240	1.82	1.48	1.0
3	10	130	320	2.75	1.95	1.5
4	15.8	180	100	21.6	7.2	7.5
5	19.3	180	24	38.9	18	21.0
6	25.4	240	32	54.6	24.1	50
7	37.1	560	42	74.1	27.5	70
7.01	37.1	560	42	114	27.5	70
8	44	750	56	91.5	35.5	105
8.01	44	750	56	125	35.5	105
9	51.4	1300	100	103.9	32.3	150
9.01	51.4	1300	100	155	32.3	150
(**a**)						
**TWR (%)**			**TW (mm^3^/min)**		***Rz* (μm)**	
Exp.^t^	Our exp.^ts^	Our numerical model	Our exp.^ts^	Our numerical model	Our exp.^ts^	Our numerical model
1	2.3	2.20	0.03	0.036	16	11.9
2	1.38	2.25	0.03	0.033	22	16.1
3	1.08	2.92	0.03	0.057	27	19.4
4	0.77	3.70	0.17	0.268	32	26.7
5	0.56	6.43	0.22	1.154	38	30.5
6	0.55	8.27	0.3	1.993	48	37.1
7 7.01	0.11	2.97	0.09	0.816	54	50.4
8 8.01	0.47	9.94	0.43	3.530	65	60.7
9 9.01	0.6	10.57	0.63	3.417	70	70.8
(**b**)						

**Table 4:** (**a**) The experimental data and the results of the electrical-thermal model with correction of multiple discharges; (**b**) The experimental data and the results of the electrical-thermal model with correction of multiple discharges.

Machining Conditions				MRR (mm^3^/min)		
Exp.^t^	Discharge current (A)	Time-on (μs)	Time-off (μs)	Our exp.^ts^	Our numerical model	Agie Sit
1	5.6	56	75	1.46	1.29	0.8
2	8.5	75	240	1.82	1.79	1.0
3	10	130	320	2.75	2.64	1.5
4	15.8	180	100	21.6	15.0	7.5
5	19.3	180	24	38.6	35.0	21.0
6	25.4	240	32	54.6	57.2	50
7 7.01	37.1 37.1	560 560	42 42	74.1 114	88.7 88.7	70 70
8 8.01	44 44	750 750	56 56	91.5 125	138 138	105 105
9 9.01	51.4 51.4	1300 1300	100 100	103.9 155	146.4 146.4	150 150
(**a**)						
TWR (%)			TW (mm^3^/min)		*Rz* (μm)	
Exp.^t^	Our exp.^ts^	Our numerical model	Our exp.^ts^	Our numerical model	Our exp.^ts^	Our numerical model
1	2.2	1.97	0.03	0.025	16	11.9
2	1.38	1.50	0.03	0.027	22	16.1
3	1.08	1.01	0.03	0.027	27	19.4
4	0.77	0.88	0.17	0.131	32	26.7
5	0.56	0.84	0.22	0.296	38	30.5
6	0.55	0.73	0.3	0.420	48	37.1
7 7.01	0.11	0.46	0.09	0.408	54	50.4
8 8.01	0.47 0.28	0.33 0.33	0.43 0.22	0.454	65	60.7
9 9.01	0.61 0.39	0.26 0.26	0.63 0.61	0.377	70	70.8
(**b**)						
